# Shopping in Reality or Virtuality? A Validation Study of Consumers’ Price Memory in a Virtual vs. Physical Supermarket

**DOI:** 10.3390/foods11142111

**Published:** 2022-07-15

**Authors:** Lina Fogt Jacobsen, Nora Mossing Krogsgaard-Jensen, Anne O. Peschel

**Affiliations:** MAPP Centre, Department of Management, Aarhus University, Fuglesangs Allé 4, 8210 Aarhus, Denmark; linaj@mgmt.au.dk (L.F.J.); noramossing@gmail.com (N.M.K.-J.)

**Keywords:** virtual reality, behavioural pricing, food choice

## Abstract

This study validates a VR supermarket as a research tool by studying the influence of the food shopping setting on consumers’ price memory—an important antecedent for price comparisons in the purchase situation. In a quasi-experiment, two groups of consumers were given a shopping task in either a physical supermarket or a virtual reality supermarket setting. Upon task completion, participants’ explicit and implicit price memory was measured across three food product categories (pizza sauce, pasta, and dark chocolate). Results revealed no significant difference between the two settings, supporting the comparability between the VR shopping experience and the experience in the physical supermarket. The VR supermarket can therefore be a valid tool for studying consumer food choice behaviour in a shopping context. Further results show that explicit price memory is weaker compared to implicit price memory, that even prices are remembered better than odd prices, and that price memory follows the expected pattern in a VR supermarket as well. Finally, exploratory findings indicate that the feeling of physical presence and self-presence is relatively high for this particular VR supermarket, whereas social presence is weaker.

## 1. Introduction

Virtual Reality (VR) is ‘*a computer-generated 3D environment—called a virtual environment—that users can navigate through and possibly interact with, resulting in real-time stimulation of one or more of the user’s five senses*’ [1], p. 2. It provides an opportunity for simulating the real-world environment [1] and is increasingly used for gaming [2], entertainment [3], education [4], therapy [5], and marketing [6,7]. In terms of research, VR can be used for studying people in an environment close to reality [8] due to its high degree of immersion and presence [9,10]. While immersion refers to the degree to which the simulation is comparable to its physical stimulus [5], presence refers to the degree to which participants feel they are an active part of the simulation [11,12].

The stunning accuracy of real-world experiences of VR simulation leads to increasing use in retailing [13,14]. This is an important development since most purchase decisions are made when consumers are in the store [15]. Moreover, retailers are increasingly competing across multiple channels [16] and on more digital dimensions [3,17], and it is predicted that retail will be one of the top industries when it comes to benefiting from VR in the nearest future). Within food, consumer purchase behaviour can also be studied using VR technologies [14] for instance in relation to healthy vs. unhealthy food purchases [18] and price interventions [19]. A typical challenge for current retail studies is the lack of control in a real store environment making the actual effects hard to predict. Moreover, the possibility of designing in-store experiments is limited by the high financial costs concerning the study design [20]. In addition, the VR setting allows for studying innovative, healthier, or more sustainable products, packaging designs, or labels, which do not yet exist in the market, with high external validity [21,22].

Several factors are linked to the intention–behaviour gap [23] in consumer purchase behaviour, including, for instance, unavailability, mistrust, and lack of knowledge [24]. One of the main barriers to consumers adopting innovative, healthier, or sustainable products is the price. While consumers usually report favourable attitudes toward these food product concepts, a large share of consumers are more reluctant to pay the premium necessary to produce these [25,26,27,28,29,30,31]. With the help of the VR supermarket, innovative, healthier, or more sustainable product concepts can be tested in a realistic setting, allowing for a more accurate prediction of what consumers might pay once the product exists on the market. Previous research found, however, that consumers are less price-sensitive in a high-immersion VR setting compared to a low-immersion VR setting [32]. On the other hand, research suggests that VR experiences can reduce hypothetical (bias in terms of willingness-to-pay compared to less immersive data collection methods [31]. Due to the relevance of price in consumer choices and the inconclusive research findings on this attribute, we investigate how prices are processed in real life compared to the VR setting by investigating consumers’ price memory on different levels [33].

Specifically, this study investigates the potential for using a VR supermarket (see introduction video https://www.youtube.com/watch?v=ztCi6otS06k, accessed on 7 June 2022) [34] for mirroring a physical supermarket when studying consumer shopping behaviour based on price memory. By comparing consumer price memory across different product categories using a VR supermarket vs. a physical (i.e., brick-and-mortar) supermarket, we can examine to what extent this tool can produce valid results reflecting realistic consumer behaviour and whether precautions should be taken when using this tool for research. Moreover, research is needed on consumer perceptions when using this type of shopping environment [1,35]. Therefore, we explore consumers’ feelings of presence in the VR supermarket.

From a research perspective, this study contributes to theory by advancing our understanding of how episodic memory in general and price memory in specific are affected by tasks performed in a virtual vs. physical environment. From a practical perspective, as the large majority of fast-moving consumer goods (FMCG) fail on the market, food practitioners may benefit from cost-effective possibilities for testing their products in a realistic environment before they are launched. In this environment, prototypes do not need to be developed before showing the product in a competitive store environment [18,22].

### 1.1. Virtual Reality Supermarkets in Research

VR supermarkets have been used for investigating, for instance, consumer attitudinal and purchase behaviour related to brands [6,8,22] and emotional responses [36]. Some studies incorporate neuroscientific tools [37] such as eye-tracking data [8,38], heart rate measurement [13], or brain activity [18]. Related to the developing possibilities for using VR to study consumer shopping behaviour [14] is the research investigating the extent to which various VR settings can be used for mirroring physical reality e.g., [19,22,39,40]. Current research shows different results, emphasising the comparability between physical and VR supermarkets and the possibility of experiencing more positive outcomes in VR compared to the physical setting [14]. For instance, Bressoud found that the comparability of attitudinal and behavioural measures between VR supermarkets and experimental physical supermarkets is questionable [22]. Pizzi, Scarpi, Pichierri and Vannucci found that consumer behaviour is comparable between VR and physical stores [1]. Siegrist, Ung, Zank, Marinello, Kunz, Hartmann and Menozzi found that food consumers’ decisions and information processing are similar between VR and physical stores [38].

One of the key features of VR is the feeling of presence that participants can experience compared to traditional experimental settings using text and/or pictures [6,40,41,42]. Presence *is ‘an illusion of “being there” in a computer-mediated environment’* [40], p. 85 and is a multi-dimensional construct consisting of perceived social presence (i.e., the experience of social actors), physical presence (i.e., the experience of physical surroundings), and self-presence (i.e., the experience of oneself) [11,43].

The feeling of presence is determined by the level of immersion (i.e., the ability of the technology to deliver an inclusive and extensive illusion of the surroundings) and interactivity (i.e., the ability of the consumer to influence the VR content during use) [2,7,32,42]. Whereas most research on VR shopping is based on lower levels of immersion, such as cave automatic VR [39] or computer-screen-based VR [19,36], Meissner, Pfeiffer, Peukert, Dietrich and Pfeiffer showed that consumers using high-immersive VR technology choose more product variety and are less price sensitive [32]. This is supported by Suh and Lee, who found that VR, compared to static picture treatment, increased the consumer’s actual and perceived product knowledge, purchase intention, and positive attitudes [44]. In a study on food consumers, Schnack, Wright and Holdershaw found that VR shopping environments with high levels of immersion increased the perceived naturalness of product interactions compared to lower levels of immersion [20]. This indicates the need for validating the specific VR technology’s ability to mirror the consumer’s actual behaviour, despite its high level of immersion, before implementing it as a research tool [37].

Several studies on VR supermarkets do not provide the possibility to interact with the products but show the products on a screen for consumers to look at [36], which has been highlighted as a disadvantage of the VR environment [22]. Other studies are based on higher interface involvement and use technology that allows consumers to interact with the material presented in terms of 3D products, such as Lee and Chung, who found that this type of VR technology increases consumer enjoyment and quality assurance, and thereby virtual presence, compared to an ordinary Internet shopping mall [40]. Likewise, Jin found that the ability to interact with 3D avatars providing marketing information influenced consumer shopping behaviour positively, primarily for consumers with low product involvement [45]. Also, Luangrath, Peck, Hedgcock and Xu found that the presence of a hand, especially in VR compared to other less interactive digital settings, increases the psychological ownership of the product because of the vicarious touch [13].

Acknowledging the importance of creating a feeling of presence when studying consumer behaviour in VR, technology in the form of head-mounted displays with handheld controllers is argued to be the most typical example of a VR technology being able to create both a high level of immersion and interactivity [2,14,32,38].

### 1.2. Price Memory as an Important Cue for Consumer Shopping Behaviour

Price is one of the most important attributes of consumer choices. Based on only two product categories, consumers tend to form a store price image [46], which is a decisive factor for store choice [47]. Behavioural pricing research suggests that consumers store observed prices along a mental number line, called the internal—or memory-based—reference price [48]. This internal reference price has been found to influence consumer choices in general [49] and specifically across store formats such as discount versus supermarket chains [50,51]. It is, therefore, of interest to analyse consumers’ price memory across store formats such as VR and a physical setting. A long tradition of consumer research shows that even though consumers tend to be price-sensitive, their price memory seems rather low [52,53]. Monroe and Lee suggest that this is not due to a consumer’s lack of knowledge but due to improperly measuring price memory [54]. They introduce the distinction between implicit memory (“knowing”) and explicit memory (“remembering”) and suggest that while explicit memory might be low, implicit memory is likely to be high. In their influential study, Vanheule and Drèze show this differentiation by measuring price memory on three dimensions: recall, recognition, and deal spotting [33]. They find that price memory improves from recall (2.1% correct) to price recognition (13.3% correct) and deal spotting (26.9% correct) suggesting that indeed implicit price memory (price recognition) is significantly higher than explicit price memory (price recall). While Vanheule and Drèze studied long-term memory prices before a shopping task [33], Jensen and Grunert adopted a similar approach to study price memory during the shopping trip [55]. They found that price memory improves during the shopping trip irrespective of price being consciously or unconsciously attended to. We base the current study on their findings by measuring implicit (price recognition) and explicit (price recall) price memory at the end of the store visit to determine whether price memory updating works similarly across the VR and physical store setting.

## 2. Materials and Methods

### 2.1. Procedure

**Figure 1:**
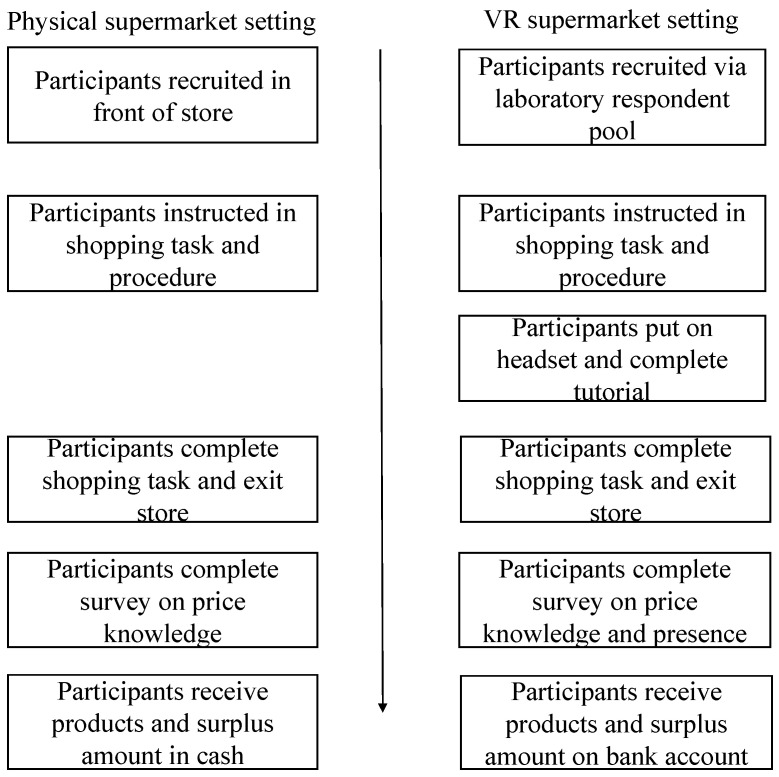
Overview of the data collection procedure.

An overview of the data collection procedure appears in Figure 1. The study was conducted as a quasi-experiment where participants participated in one of two settings: a shopping task in a VR or a physical supermarket. In both settings, participants were instructed to buy one product from each of three product categories (pizza sauce, pasta, and dark chocolate). These categories were selected due to their regular consumption by Danish consumers. The participants were told to form an overall impression of the product range available on the shelves within each category, choose the product they favoured most from each, and then buy them. In the physical supermarket, each participant received around 20 euros (150 DKK). They got to keep the purchased products and the rest of the money. In the VR supermarket, participants were given the same amount of money, and by the end of the study, they received the selected products (in physical form), and the rest of the money was transferred to their bank account. Both conditions were incentive compatible, in that participants had to trade off their perceived product value and the observed price to maximize their expected earnings regarding the products received and their monetary payment. In both settings, the participants were asked to fill out a survey immediately after the shopping task was completed. In the physical supermarket setting, they answered via an iPad, whereas the survey was completed on a stationary computer in the VR supermarket setting after removing the headset and controllers.

The first questions addressed the participants’ price recall and price recognition (for two randomly selected products of the three). For the VR supermarket setting, the price memory questions were followed by questions on the feeling of presence in the VR setting.

### 2.2. Virtual Reality

**Figure 2:**
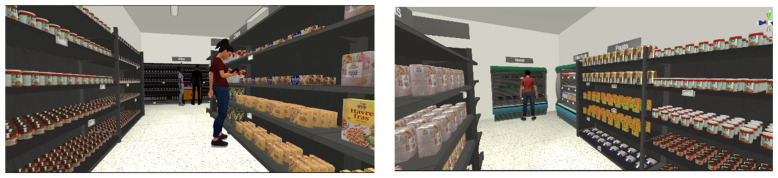
Pictures showing an example of the participant perspective in the VR supermarket.

The HP Reverb G2 Omnicept Edition headset was used in the VR supermarket setting. This high-resolution headset allows 114 degrees of viewing at 2160 × 2160 pixels per eye with a refresh rate of 90 Hz. The supermarket setting (Figure 2) was developed in Unity (version 2020 3.13f1). The setting consisted of a rectangular store with two main aisles and shelves stacked with interactable 3D models of products from different FMCG categories such as toilet paper, wine, pasta, and crisps. The setting also contained a cooler for storing meat and dairy products. All products were modelled based on common products found in the Danish market. Moreover, to provide a realistic setting and an overview of the store, the setting included store background noise, product signs, other customers (non-interactive), a cashier, and staff. By using the handheld controllers, participants were able to pick up and rotate all products to read the package description and place them in the basket. Buttons were used to interact with the products and place them in the basket. The high resolution allowed participants to easily read the product descriptions on the packaging and price information on the shelves. Participants could physically move closer to shelves, but to ensure their safety, teleportation was primarily used for navigating around the store. All participants were given a tutorial familiarising themselves with teleportation and product interaction, including pick-up of products, rotating and inspection, and placing them in the basket. Prior to the study, participants were informed about the possible experience of feeling dizzy during the task and, in that case, contacting the research assistant immediately to cancel participation. No participants reported experience of sickness or dizziness. No time restrictions were placed on the shopping task.

### 2.3. Participation

Within each setting, the final pool of participants varied in age (mean_physical_ = 32 ranging from 18 to 82 years, mean_VR_ = 26 ranging from 18 to 79 years, t-value_age_ = 2.31, *p* < 0.05) and gender (female_physical_ = 38%, male_physical_ = 60%, other_physical_ = 2%, female_VR_ = 44%, male_VR_ = 56%, χ^2^-value_gender_ = 1.75, *p* > 0.05), with a fair spread in distribution of age and gender across the two settings. The participants in the physical supermarket setting (n = 50) were recruited in a large shopping centre in Aarhus, Denmark, close to the university laboratory, in front of a large, well-known Danish supermarket. Potential participants were approached by the researchers and invited to participate in a study on purchase behaviour. In the VR supermarket setting, participants (n = 52) were recruited from a participant pool related to a university laboratory. The participant pool consists of students and persons from other demographics recruited at local fairs and festivals. Participants could sign up for different time slots, and data were collected in the laboratory. Researchers instructed participants on how to use the headset, and each of them were given a tutorial in the VR setting before the actual study took place. The tutorial trained the participants in moving around the store and using the handheld controllers for picking up, rotating, and purchasing products. To avoid biased results, the price was mentioned in neither of the settings in the information to participants. In both settings, the data collection took place at different times of day (morning, midday, and evening), during both weekdays and weekends.

### 2.4. Measures

Due to the implicit and explicit measures of price memory [33], this study measured price memory in two ways: price recall implies that the consumer knows the price “by heart” and was measured by asking participants to write the actual price of the product. To compare across product categories, the absolute percentage difference was calculated [33]. Price recognition implies that the actual price cannot be remembered without any references, but when shown, consumers can tell whether it is the correct price. This was measured by asking participants to choose the correct price among three possible answers (the correct price, 10% above, and 10% below). Due to an unexpected sale for the chocolate category in the physical supermarket, price recognition was not measured in this category.

The feeling of presence was measured along three constructs: perception of physical presence, social presence, and self-presence to capture immersion and interactivity (see Table 3 for an overview). All constructs were treated as latent constructs and measured with multiple items on a 5-point Likert scale based on Makransky, Lilleholt and Aaby [11]. Physical presence was measured with five items. Social presence was measured with three items. Two items from the original scale were removed as they focused explicitly on interaction with other people in the store, which was not the focus of our research design. Self-presence was measured using four items. Again, one item was removed from the original scale as it did not fit our research design.

## 3. Results

### 3.1. Descriptive Results and Comparison of Price Memory between Settings

**Figure 3:**
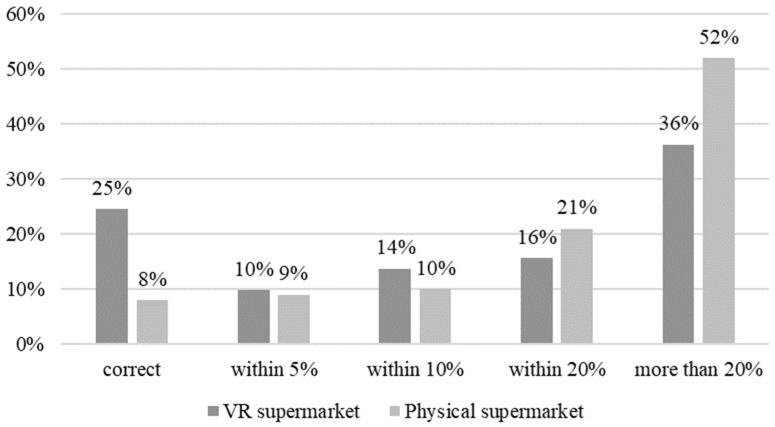
Price recall across product categories at different levels of accuracy.

Overall, participants display a relatively low explicit price memory across product categories and store formats. The average error was 19% in the VR supermarket and 24% in the physical supermarket. Figure 3 shows that more than one-third of the participants in the VR setting and more than half in the physical setting were more than 20% off with their price estimates. The difference in correctly recalled prices between store formats was mainly due to chocolate being recalled correctly by 51% of participants in the VR setting (Table 1). For the other categories, the patterns are very similar across store settings.

An independent sampled *t*-test was conducted to compare the deviations (in numerical value) between the actual price, and the individual price recalls for each product category between the physical and the VR supermarket setting. The results are insignificant (*t*-value_pasta_ = 1.13, *p*-value_pasta_ = 0.26, *t*-value_pizza sauce_ = 1.56, *p*-value_pizza sauce_ = 0.12, *t*-value_chocolate_ = 0.83, *p*-value_chocolate_ = 0.41). This implies no difference in the participant’s ability to recall prices between the store formats for the three product categories. Moreover, it indicates a relatively low explicit price memory for pasta and pizza sauce, whereas this is higher for the chocolate product category, which was even-priced and on sale.

Table 2 shows the percentage distribution of the participant’s ability to recognise the actual price paid for each of the three products. Due to an unexpected sale of chocolate in the physical store, the results are only available for the pasta and pizza sauce product categories.

Again, an independent sampled *t*-test was conducted to compare the participant’s ability to recognise prices for the products (pizza and pasta) between the physical and VR supermarket setting. Since the three possible answers were −10% of the actual price, actual price, and +10% of actual price) the dependent (recognition) variable was treated as numeric. The results are insignificant (n_pizza sauce,VR_ = 27, n_pizzasauce,physical_ = 38, *t*-value_pizzasauce_ = 0.35, *p*-value_pizzasauce_ = 0.73, n_pasta,VR_ = 40, n_pasta,physical_ = 39, *t*-value_pasta_ = −1.32, *p*-value_pasta_ = 19). This implies that there is no difference in the participants’ ability to recognise prices between the two store formats for the two product categories.

### 3.2. Measurement Model and Descriptive Statistics of Consumer Perceived Presence in the VR Supermarket

A confirmatory factor analysis (CFA) based on the maximum likelihood estimator [56] was conducted to assess the reliability and validity of the multi-item scales [57]. The analysis was conducted in Amos28. Due to low or insignificant factor loadings, all social presence items were removed from the CFA and treated as individual variables. Supporting the convergent validity, the model shows an acceptable fit (NFI = 0.899) with all factor loadings 0.5 or higher. Composite reliability (CR) and average variance extracted (AVE) are above the suggested cut-off levels of 0.8 and 0.6, except for AVE regarding physical presence. However, given the acceptable factor loadings and their inclusion in the original scale [11], we decided to keep all items of the construct (Table 3). The correlation between the two constructs was high (0.90). Again, as they are both part of the validated scale for the higher-order presence-construct, this is not surprising, but an interpretation of the two constructs for further analyses should consider this.

The descriptive statistics (Table 3) show that the mean physical presence and self-presence are above 3 on the 5-point Likert scale. Despite not being able to compare these measures with perceived presence in other environments, the results indicate a generally positive experience regarding the feeling of presence among participants in the VR supermarket setting. The social presence dimension is more problematic since all item means are on average perceived lower than 3, indicating that the feeling of social presence is relatively low compared to the other presence dimensions.

## 4. Discussion

The main goal of this study was to compare shopping experiences in a physical and a VR supermarket by identifying differences in consumers explicit and implicit price memory. The objective was twofold; (1) to contribute to the academic literature on behavioural pricing and reference price, and (2) to validate a VR supermarket for research purposes. We, therefore, conducted a quasi-experiment where participants were asked to shop for products in three food product categories in either the VR supermarket or the physical supermarket setting. In addition, presence was measured to account for participants’ immersion and interactivity in the VR setting.

### 4.1. Price Memory across Store Formats

The results show that consumers’ price memory does not differ across the store formats. This finding might be surprising since VR supermarkets are typically smaller and consist of fewer products and shelves [14], which is also the case in this study. One may argue that consumers would find it easier to remember prices when the products are fewer. However, even though the range of products is broader in the physical supermarkets, prices are often the same for variants of the same brand, so the number of prices to be remembered may not differ much in the end. In fact, in our study, explicit price memory for chocolate, pasta, and pizza sauce was low, which is in line with prior research [33,52,53]. The average error for price recall estimates was comparable to those previously assessed [33,52], suggesting that the settings in our research were similar to other previously studied purchase situations. Implicit price memory for pizza sauce and pasta was higher but still showed a considerable deviation both upward and downward from the correct price. In line with Schindler and Wiman, explicit price memory for chocolate was considerably higher in both store settings due to the product being on sale [58] and, therefore, evenly priced. Importantly, there was no difference in price memory between the two store settings for any product categories. We conclude that the shopping experience in the VR supermarket is comparable to that of the physical supermarket. This is in line with other research on consumer behaviour in a VR setting [1,38]. In addition, we do not find support for consumers being less price-sensitive in the high-immersive VR settings [32], as both implicit and explicit price knowledge are indifferent at the store exit. Notably, in Meissner et al. (2020), participants were found to be less price-sensitive in the high-immersion compared to a low-immersion condition. The physical supermarket that we compare in our study could be considered another high-immersion setting, which supports our findings of no difference in price-memory across high-immersion setting. It should also be noted that we immersed participants in a whole VR supermarket and no only a VR shelf [32], which should contribute to creating an experience that can *substitute perceived reality* [14]. That sales prices were remembered better in both store settings supports our assumption that the participants are equally invested in product prices across the two settings.

Overall, our results lend support to previous research on price memory. Explicit price memory is weaker than implicit price memory, and even prices are remembered better than odd prices, including in a VR supermarket. Overall, these findings align with Fang, Nayga Jr., West, Bazzani, Yang, Lok, Levy and Snell) in that the high-immersion VR experience should reduce hypothetical bias [31].

### 4.2. The Feeling of Presence in VR Supermarket

Our research presents positive findings regarding the feeling of presence when consumers are shopping in the virtual supermarket, which aligns with the suggestion of using a head-mounted display together with handheld controllers as a tool supporting the feeling of presence based on immersion and interactivity [32,38]. This supports the VR supermarket’s ability to create an illusion of being in the store, which is a key feature of conducting research in this setting.

Physical presence, which reflects the participant’s experience of the physical surroundings, is relatively high, indicating that the level of immersion is good. While most VR-based research in consumer behaviour uses environments with lower levels of immersion [19,32], our VR supermarket provides an opportunity for conducting studies with high levels of immersion improving the feeling of physical presence. Moreover, the results on self-presence, which reflects the experience of oneself being part of the environment, indicate that participants largely feel part of the VR supermarket. Using handheld controllers where participants can pick up and rotate products to inspect them further can be a reason for this feeling. This possibility allows them to influence the VR content, which is the key aspect of interactivity [7,32]. As several studies in existing literature do not allow for this possibility to interact with, for instance, products [22,36], it is positive to have a VR supermarket where consumer feelings of self-presence are improved based on the option to use their hands (via handheld controllers) for inspecting products, as they would do in a physical supermarket.

Finally, whereas the feeling of physical presence and self-presence seems to be at satisfactory levels, indicating the overall feeling of “being there”, the results show that the feeling of social presence, which is the participants’ experience of the social actors in the VR environment, is relatively low compared to the two other presence constructs. Our VR supermarket did have a salesperson and other customers in the form of avatars in the store (Figure 2), but participants perceived them as less aware, conscious, or alive. This could be explained by the research design where the focus was on participants interacting with products and moving around the store environment and not on interaction with staff or other customers. Instead, the avatars mainly functioned as part of the background for creating a realistic store atmosphere.

## 5. Conclusions

This study contributes to the emerging stream of research on VR shopping as it validates the chosen VR supermarket as a research tool by studying the influence of the food shopping setting on consumer price memory. Specifically, both implicit and explicit price memory in the physical supermarket is well reflected in the VR supermarket, suggesting comparable shopping experiences across settings. Moreover, the exploratory results on perceived presence indicate a satisfactory level of both physical and self-presence, whereas social presence is weaker. Overall, the study supports the use of this highly immersive and interactive VR supermarket for future research on consumers in-store shopping behaviour for food products.

## 6. Limitations and Future Research

This study validates this VR supermarket for research purposes. Despite positive results on the comparability between the VR and the physical setting on the price memory and perceived presence, validation studies on other factors, such as mode of interaction, movement, and range of product categories influencing consumer shopping behaviour should be considered. Although existing studies show promising results of using VR in food and retail, the specific technologies must always be validated [37].

Moreover, the validity of a VR supermarket may depend on the specific products under investigation [44]. For some products, consumers may find it more relevant to pick up and rotate them for inspecting the packaging (which is possible in this VR supermarket). In contrast, other products may require another type of inspection (e.g., smelling fresh fruit or meat).

Regarding the sampling, the data shows a fair spread of age and gender across the two settings, which supports the validity of the results. Yet, participants in the VR setting were significantly younger compared to the physical supermarket setting. This is a disadvantage of the chosen quasi-experimental design, which was intended to capture least bias in shopping behaviour by observing shoppers in their physical supermarket and lab participants in the VR setting. We assume memory processes for healthy adults to work similarly, irrespective of age, and can draw valid inferences from this study. However, future research could consider other individual characteristics which may influence consumer shopping behaviour related to, for instance, socioeconomic status or cultural differences [14]. Another individual consumer characteristic that could be important is technology innovativeness or readiness, as this has been found to influence consumer acceptance of new technologies [59].

Focusing on the construct of presence, a validated scale was used [11] to capture the three dimensions: physical presence, self-presence, and social presence. However, the CFA showed some limitations in the measures, especially on social presence, but also regarding the other two dimensions (relatively low NFI, high correlation between constructs, and low AVE for physical presence). Results on presence as an overall construct should therefore be interpreted carefully. Acknowledging this limitation in our research, we have reported the individual items (Table 3). Future research could aim to increase the validity of the presence measure by including alternative presence scales.

Finally, the social presence validity needs to be explored further. This study did not focus on social interaction, and despite simulating other customers and staff in the VR supermarket (Figure 2), participants could not interact with them. Future studies can investigate the influence of social interaction with other customers, or different levels of crowding in the store on a consumer’s shopping experience [3] as the presence of others can significantly influence consumer shopping behaviour [14]. More research is needed to optimally simulate this social factor in the shopping situation in future studies [33].

## Figures and Tables

**Table 1:** Percentages of participants’ ability to recall the actual price for each product category.

	Physical Supermarket	VR Supermarket
Pasta	0%	7%
Pizza sauce	3%	7%
Chocolate	30%	51%

**Table 2:** Percentages of the participant’s ability to recognise the actual price paid for the products.

		Percentages of Participants	
	Presented Price Relative to Actual Price	PASTA	PIZZA Sauce
Physical supermarket	−10%	38%	29%
	Actual price	31%	34%
	+10%	31%	37%
VR supermarket	−10%	40%	19%
	Actual price	50%	48%
	+10%	10%	33%

**Table 3:** Constructs, measurement scales, and descriptive statistics.

Constructs and Items	Mean (SD)	Λ	^b^ C.R.	^c^ AVE
*Physical presence*	3.85 (0.71)		0.790	0.45
The virtual environment seemed real to me.	3.77 (0.94)	0.558 ***		
I had a sense of acting in the virtual environment rather than operating something from the outside.	4.40 (0.77)	0.600 ***		
My experience in the virtual environment seemed consistent with my experiences in the real world.	3.19 (1.10)	0.496 **		
While in the virtual environment, I had a sense of “being there”.	4.04 (0.91)	0.751 ***		
I was completely captivated by the virtual world.	3.83 (1.01)	0.846 ***		
*Social presence*	-		-	
^a^ I felt I was in the presence of another person in the virtual environment.	2.21 (1.07)	-		
^a^ I felt that the people in the virtual environment were aware of my presence.	1.77 (0.81)	-		
^a^ The people in the virtual environment appeared to be sentient (conscious and alive) to me.	2.35 (1.12)	-		
*Self-presence*	3.51 (0.98)		0.881	0.65
I felt like my virtual embodiment was an extension of my real body within the virtual environment.	3.56 (1.24)	0.770 ***		
I felt like my real hand was projected into the virtual environment through my virtual embodiment.	3.77 (1.10)	0.716 ***		
I felt like my real hand was inside the virtual environment.	3.17 (1.25)	0.893 ***		
During the simulation, I felt like my virtual embodiment, and my real body became one and the same.	3.54 (1.10)	0.834 ***		

** *p*-value < 0.01 *** *p*-value < 0.001 ^a^ item removed due to low or insignificant factor loadings. ^b^ CR = $\frac{{\left(\sum_{i=1}^{n}L_{i}\right)}^{2}}{{\left(\sum_{i=1}^{n}L_{i}\right)}^{2}+{\left(\sum_{i=1}^{n}e_{i}\right)}^{}}$ ^c^ AVE = $\frac{\sum_{i=1}^{n}\lambda_{i}^{2}}{n}$.

## Data Availability

The data presented in this study are available on request from the corresponding author. The data are not publicly available due to privacy.

## References

[1] Pizzi G., Scarpi D., Pichierri M., Vannucci V. (2019). Virtual reality, real reactions?: Comparing consumers’ perceptions and shopping orientation across physical and virtual-reality retail stores. Comput. Hum. Behav..

[2] Suh A., Prophet J. (2018). The state of immersive technology research: A literature analysis. Comput. Hum. Behav..

[3] Van Kerrebroeck H., Brengman M., Willems K. (2017). Escapring the Crowd: An experimental study on the impact of a virtual reality experience in a shopping mall. Comput. Hum. Behav..

[4] Johnson-Glenberg M.C. (2018). Immersive VR and Eduation: Embodied Design Principles That Include Gesture and Hand Controls. Front. Robot. AI.

[5] Bowman D.A., Mcmahan R.P. (2007). Virtual Reality: How Much Immersion is Enough?. Computer.

[6] Van Kerrebroeck H., Brengman M., Willems K. (2017). When Brands Come to Life: Experimental research on the vividness effect of virtual reality in transformational marketing communications. Virtual Real..

[7] Wedel M., Bigné E., Zhang J. (2020). Virtual and augmented reality: Advancing research in consumer marketing. Int. J. Res. Mark..

[8] Bigné E., Llinares C., Torrecilla C. (2016). Elapsed time on first buying triggers brand choices within a category: A virtual reality-based study. J. Bus. Res..

[9] Crofton E., Murray N., Botinestean C. (2021). Explroing the Effects of Immersive Virtual Reality Environments on Sensory Perception of Beef Steaks and Chocolate. Foods.

[10] Oliver J.H., Hollis J.H. (2021). Virtual Reality as a Tool to Stucy the Influence of the Eating Environment on Eating Behavior: A feasibility study. Foods.

[11] Makransky G., Lilleholt L., Aaby A. (2017). Development and Validation of the Multimodel Presence Scale for Virtual Reality Environments: A confirmatory factor analysis and item response theory approach. Comput. Hum. Behav..

[12] Steuer J. (1992). Defining Virtual Reality: Dimensions Determining Telepresence. J. Commun..

[13] Luangrath A.W., Peck J., Hedgcock W., Xu Y. (2021). Observing Product Touch: The Vicarious Haptic Effect in Digital Marketing and Virtual Reality. J. Mark. Res..

[14] Xi N., Hamari J. (2021). Shopping in Virtual Reality: A literature review and future agenda. J. Bus. Res..

[15] Inman J.J., Winer R.S., Ferraro R. (2009). The Interplay among Category Characteristics, Customer Characteristics, and Customer Activities on in-Store Decision Making. J. Mark..

[16] Pantano E., Viassone M. (2015). Engaging consumers on new integrated multichannel retail settings: Challenges for retailers. J. Retail. Consum. Serv..

[17] Vrechopoulos A.P., O’Keefe R.M., Doukidis G.I., Siomkos G.J. (2004). Virtual store layout: An experimental comparison in the context of grocery retail. J. Retail..

[18] Ruppert B. (2011). New Directions in the Use of Virtual Reality for Food Shopping: Marketing and Education Perspectives. J. Diabetes Sci. Technol..

[19] Waterlander W., Scarpa M., Lentz D., Steenhuis I.H.M. (2011). The virtual supermarket: An innovative research tool to study consumer food purchasing behaviour. BMC Public Health.

[20] Schnack A., Wright M.J., Holdershaw J.L. (2019). Immersive Virtual Reality Technology in a Three-Dimensional Virtual Simulated Store: Investigating telepresence and usability. Food Res. Int..

[21] Harz N., Hohenberg S., Homburg C. (2021). Virtual Reality in New Product Development: Insights from Prelaunch Sales Forecasting for Durables. J. Mark..

[22] Bressoud E. (2013). Testing FMCG innovations: Experimental real store versus virtual. J. Prod. Brand Manag..

[23] Ajzen I., Brown T.C., Carvajal F. (2004). Explaining the Discrepancy between Intentions and Actions. Pers. Soc. Psychol. Bull..

[24] Qi X., Yu H., Ploeger A. (2020). Exploring Influential Factors Including COVID-19 on Green Food Purchase Intentions and the Intention–Behaviour Gap: A Qualitative Study among Consumers in a Chinese Context. Int. J. Environ. Res. Public Health.

[25] Aschemann-Witzel J., Zielke S. (2015). Can’t Buy Me Green? A Review of Consumer Perceptions of and Behavior Toward the Price of Organic Food. J. Consum. Aff..

[26] Peschel A.O., Aschemann-Witzel J. (2020). Sell more for less or less for more? The role of transparency in consumer response to upcycled food products. J. Clean. Prod..

[27] Aschemann-Witzel J., Varela P., Peschel A.O. (2019). Consumers’ categorization of food ingredients: Do consumers perceive them as ‘clean label’ producers expect? An exploration with projective mapping. Food Qual. Prefer..

[28] Marian L., Chrysochou P., Krystallis A., Thøgersen J. (2014). The role of price as a product attribute in the organic food context: An exploration based on actual purchase data. Food Qual. Prefer..

[29] Peschel A., Grebitus C., Steiner B., Veeman M. (2016). How does consumer knowledge affect environmentally sustainable choices? Evidence from a cross-country latent class analysis of food labels. Appetite.

[30] Hofstetter R., Miller K.M., Krohmer H., Zhang Z.J. (2013). How Do Consumer Characteristics Affect the Bias in Measuring WIllingness to Pay for Innovative Products?. J. Prod. Innov. Manag..

[31] Fang D., Nayga R.M., West G.H., Bazzani C., Yang W., Lok B.C., Levy C.E., Snell H.A. (2021). On the Use of Virtual Reality in Mitigating Hypothetical Bias in Choice Experiments. Am. J. Agric. Econ..

[32] Meissner M., Pfeiffer J., Peukert C., Dietrich H., Pfeiffer T. (2020). How Virtual Reality Affects Consumer Choice. J. Bus. Res..

[33] Vanhuele M., Drèze X. (2002). Measuring the Price Knowledge Shoppers Bring to the Store. J. Mark..

[34] (2022). VR-Supermarket. https://food.au.dk/foodhay/instruments/sensory-and-consumer-platform/multisensory-biometrics-lab/vr-supermarket.

[35] Lau H.F., Kan C.W., Lau K.W. (2013). How Consumers Shop in Virtual REality? How it works?. Adv. Econ. Bus..

[36] Massara F., Liu S.S., Melara R.D. (2010). Adapting to a retail environment: Modeling consumer–environment interactions. J. Bus. Res..

[37] Dijksterhuis G., De Wijk R.A., Onwezen M. (2022). New Consumer Research Technology for Food Behaviour: Overview and validity. Foods.

[38] Siegrist M., Ung C.-Y., Zank M., Marinello M., Kunz A., Hartmann C., Menozzi M. (2019). Consumers’ food selection behaviors in three-dimensional (3D) virtual reality. Food Res. Int..

[39] Van Herpen E., van den Bruek E., van Trijp HC M., Yu T. (2016). Can a Virtual Supermarket Bring Realism Into the Lab? Comparing shopping behavior using virtual and pictorial store representations to behavior in a physical store. Appetite.

[40] Lee K.C., Chung N. (2008). Empirical analysis of consumer reaction to the virtual reality shopping mall. Comput. Hum. Behav..

[41] Nichols S., Haldane C., Wilson J.R. (2000). Measurement of presence and its consequences in virtual environments. Int. J. Human-Computer Stud..

[42] Schuemie M.J., Van Der Straaten P., Krijn M., Van Der Mast C.A.P.G. (2001). Research on Presence in Virtual Reality: A survey. CyberPsychol. Behav..

[43] Lee K.M. (2014). Presence, Explicated. Commun. Theory.

[44] Suh K.S., Lee Y. (2005). The Effects of Virtual Reality on Consumer Learning: An empirical investigation. MIS Q..

[45] Jin S.A. (2009). The Roles of Modality Richness and Involvement in Shopping Behavior in 3D Virtual Stores. J. Interact. Mark..

[46] Ofir C., Raghubir P., Bosh G., Monroe K.B., Heiman A. (2008). Memory-Based Store Price Judgements: The role of knowledge and shopping experience. J. Retail..

[47] Hamilton R., Chernev A. (2013). Low Prices are Just the Beginning: Price Image in Retail Management. J. Mark..

[48] Cheng L.L., Monroe K.B. (2013). An appraisal of behavioral price research (part 1): Price as a physical stimulus. AMS Rev..

[49] Mazumdar T., Raj S.P., Sinha I. (2005). Reference Price Research: Review and Propositions. J. Mark..

[50] Elshiewy O., Peschel A.O. (2021). Internal reference price response across store formats. J. Retail..

[51] Koschmann A., Isaac M.S. (2018). Retailer Categorization: How Store-Format Price Image Influences Expected Prices and Consumer Choices. J. Retail..

[52] Dickson P.R., Sawyer A.G. (1990). The Price Knowledge and Search of Supermarket Shoppers. J. Mark..

[53] Gabor A., Granger C.W.J. (1961). In the Price Consciousness of Consumers. J. R. Stat. Soc. Ser. C (Appl. Stat.).

[54] Monroe K.B., Lee A.Y. (1999). Remembering versus Knowing: Issues in Buyers’ Processing of Price Information. J. Acad. Mark. Sci..

[55] Jensen B.B., Grunert K.G. (2014). Price Knowledge during Grocery Shopping: What We Learn and What We Forget. J. Retail..

[56] Arbuckle J.L. (2014). IBM SPSS Amos 23 User’s Guide.

[57] Hair J.F., Black W.C., Babin B.J., Anderson R.E. (2021). Multivariate Data Analysis: A Global Perspective.

[58] Schindler R.M., Wiman A.R. (1989). Effects of odd pricing on price recall. J. Bus. Res..

[59] Lin C.H., Shih H.Y., Sher P.J. (2007). Integrating Technology Readiness into Technology Acceptance: The TRAM model. Psychol. Mark..

